# The self-relevant spotlight metaphor: Self-relevant targets diminish distractor–response-binding effects

**DOI:** 10.3758/s13423-024-02455-x

**Published:** 2024-11-18

**Authors:** Marcel Pauly, Sarah Schäfer, Dirk Wentura, Christian Frings

**Affiliations:** 1 Department of Psychology, Saarland University, Campus A2 4, 66123 Saarbrücken, Germany; 2 University of Trier, Trier, Germany

**Keywords:** Self-relevance, Cognitive control, Executive control, Distractor–response binding

## Abstract

Recently, it has been proposed that self-relevance of a stimulus enhances executive control and reduces the impact of distractors on current task performance. The present study aimed to test whether the binding between a distractor and a response is influenced by self-relevance, too. We assumed that targets’ self-relevance should increase executive control processes and therefore reduce the influence of distractors on current performance. In a distractor–response-binding (DRB) task, which measures the strength of binding between distractor stimuli and responses, we varied target relevance so that participants responded to targets that either were or were not self-relevant. Our design made it possible to measure DRB effects for both relevance conditions separately. DRB effects were diminished if targets were self-relevant compared to when they were not. These results expand our understanding of the influence of self-relevance on cognitive performance. The influence of self-relevance is not purely perceptual (Sui & Humphreys, 2012, *Journal of Experimental Psychology: Human Perception and Performance, 38*[5], 1105–1117), but also found in higher-order processes such as executive control. Moreover, whereas for different paradigms binding advantages of self-relevance are assumed (Sui & Humphreys, 2015a, *Trends in Cognitive Sciences*, *19*[12], 719–728; Humphreys & Sui, 2016, *Cognitive Neuroscience, 7*[1/4], 5–17), this study identifies an important boundary condition, in that distractor–response binding is reduced by target self-relevance.

Distracting stimuli are everywhere in the environment that surrounds us. When pursuing an action goal, stimuli that do not contribute to this goal or even interfere with it can (at least temporarily) be seen as distractors. In many theories of attention and executive control, it is assumed that not only are targets amplified, but distractors are suppressed in order to achieve goal-directed behaviour (Gaspelin & Luck, 2018; Neumann & DeSchepper, 1991; Sawaki & Luck, 2010; Tipper & Cranston, 1985). Yet, irrespective of whether a distractor is suppressed, it can still impact upon action control via binding and retrieval (Frings, Rothermund, et al. 2007; Frings et al., 2020). Giesen et al. (2012) found that in a distractor–response binding task, distractors are bound to target responses; thus, subsequent presentation of the distractor stimuli can cause retrieval of these target responses irrespective of the amount of interference the distractors could elicit during prime integration. This can be advantageous if irrelevant features have a predictive effect on the occurrence of relevant features; hence it seems functional that our cognitive system exploits these contingencies via distractor-based retrieval mechanisms (Miller, 1987; Wiswede et al., 2013).

Distractor processing can be modulated by several aspects. There are numerous instances in the literature that show that distractor processing and distractor suppression, in particular, are modulated by context. For example, valent distractors might attract more attention than non-valent distractors and are harder to ignore (Frings, Rothermund, et al., 2007; Frings, Wentura, et al. 2007; Wentura et al., 2013). Distractors that are associated with current concerns or needs receive more attention and again are harder to ignore (Frings, 2006), whereas distractors presented at predictable locations are easier to ignore (Wang & Theeuwes, 2020). Stress, on the other hand, can make target selection more effective, such that distractors lose their ability to draw attention (Chajut & Algom, 2003; Frings et al., 2013).

Here we focus on a particularly powerful modulating context factor, namely self-relevance. In the recent literature, it has been suggested that self-relevance has an impact on selection processes by enhancing executive control (Golubickis & Macrae, 2023; Svensson et al., 2023). Specifically, in a series of Stroop experiments, Dignath et al. (2022) found less interference from response-incompatible stimuli if participants were primed with self-relevant stimuli. In another study it could be shown that persons can inhibit a response to a self-relevant item (operationalized by whether an item belongs to the person himself or to another person) better after a stop signal than to a non-self-relevant item (Golubickis et al., 2021). Also, Golubickis and Macrae (2021) found that self-relevant targets cancelled out the impact of distractors entirely. In their experiment, participants were presented with an item deemed to be “owned” by them and an item deemed to be “owned” by a friend; their task was to judge whether target stimuli belonged to them or the friend. These target stimuli were surrounded by distractors that were either compatible with the target (i.e., distractors and targets were identical), neutral (the distractor was not owned by either person) or incompatible (the distractor was owned by a person who does not own the target). They found a flanker-interference effect when the target was related to the other person, but no such effect when the target was self-relevant. In other words, (incompatible) flanking distractors just had an influence when the targets were not self-relevant. The overarching conclusion drawn from this research is that self-relevance can influence top-down mechanisms like executive control processes.

Given this backdrop, we were interested in whether distractor–response binding (DRB; Frings, Rothermund, et al. 2007a) is modulated by self-relevance. The DRB task is a paradigm rooted in action control research. It has been suggested that in sequential tasks that have a prime-probe structure, features of all stimuli (including distractors) are bound to the response (the *binding* part, resulting in an event file; Frings et al., 2020). Then, if a feature is repeated in the next display, the entire previous episode or event file is retrieved (the *retrieval* part), which in turn modulates response execution. If a distractor is repeated in a sequential paradigm, it facilitates performance if the required response is repeated as well, but hampers performance if the response changes between prime and probe displays. Similarly, when a distractor changes between prime and probe displays, performance is facilitated when the required response changes, too. It is hampered if the response has to be repeated.

The *Binding and Retrieval in Action Control* framework (BRAC; Frings et al., 2020), assumes that binding and retrieval can be separately influenced by top-down and bottom-up mechanisms (see also Hommel & Wiers, 2017), which also applies for distractor–response binding. Several studies have yet shown an influence of bottom-up processes on distractor–response binding. For example, grouping by color (Laub et al., 2018) or by stimulus intensity (Laub & Frings, 2021) impacts distractor binding. When distractors and targets are grouped, distractor integration is enhanced. Recently, it was also shown that figure-ground mechanisms influence DRB effects (Schmalbrock & Frings, 2022).

We propose that self-relevance is an additional factor that will have an impact on DRB, assuming that self-relevance increases executive control and thereby diminishes the impact of distractors in a top-down manner. If a target is self-relevant, distractor processing should decrease, which in turn should result in the reduction (or even absence) of DRB effects.

We run a variant of the DRB task in which participants responded to pronouns that were either self-relevant (e.g., “I”; “myself”) or not self-relevant (e.g., “he”; “she”). Pronouns were presented on shapes that were irrelevant to the task—these were the distractors. We used eight different pronouns (four of them self-relevant) mapped onto four response keys. Our assignment of pronouns to response keys made it possible to orthogonally vary distractor and response repetition between prime and probe separately within each (self-)relevance condition. In other words, the paradigm allowed us to calculate DRB effects independently for the two relevance conditions (i.e., self vs. other). We expected to find diminished DRB effects for the condition with self-relevant targets.

## Method

Raw data and analysis scripts for the experiment can be found at https://osf.io/3gkrp.

### Participants

With regard to power planning, we consulted Schmalbrock and Frings (2022). In their study, the average moderation of DRB effects across three experiments was *d*_*Z*_ = 0.49, but we decreased our expectation to *d*_*Z*_ = 0.40. To find an effect of *d*_*Z*_ = 0.40, given α = .05 (two-tailed), with a power of 1 – β = .80, requires a minimum sample size of 52 participants (calculated with G*Power; Faul et al., 2009).

Participants took part in exchange for course credit. In total, we collected the data of 64 participants to account for possible drop-outs. Some participants were excluded from the analysis because they answered at chance level (error rates were >72%; *n* = 3), because they did not follow the instructions correctly and we were therefore not able to calculate DRB effects (*n* = 4), or because their overall RTs were identified as extreme (i.e., 3 interquartile ranges beyond the third quartile; Tukey, 1977; *n* = 3). Our final sample thus comprised *N* = 54 participants. Median age was 22 years (ranging from 19 to 38), 29 participants were female, and participants had normal or corrected-to-normal vision.

### Design

The experiment used a 2 (response relation: *repetition* vs. *change*) × 2 (distractor relation: *repetition* vs. *change*) × 2 (relevance: *self* vs. *other*) within-participants design.

### Material and apparatus

The experiment was built with PsychoPy3 and the PsychoJS library (Peirce et al., 2019). The study was conducted online at pavlovia.org. Participants were allowed to use a desktop or laptop computer to take part; operating systems used were Windows (*n* = 44) and MacOS (*n* = 10), and browsers used were Google Chrome (*n* = 28), Mozilla Firefox (*n* = 17), and Safari (*n* = 9). All screens had a refresh rate of 60 Hz. Display resolutions ranged from 1200 × 800 to 1280 × 720 to 2560 × 1440. Actual size of stimuli on-screen was not assessed.

All target words were presented in white Arial font in the center of the screen. We used the German words *ich* [I], *mich* [myself], *mir* [me] and *mein* [my] as self-relevant terms, and *er* [he], *sie* [she], *ihr* [(the plural) you] and *euer* [(the plural) your] as non-self-relevant terms. The distractor shapes (triangle, cross, square, or circle) were blue (RGB values [47, 82, 143]) and always presented behind the target words. The background was black. Letter height was set to 5 % of the display height (this also defined letter width as the font aspect ratio was retained). Distractor height and width were set to 10 % of the display height. The height and width of an asterisk that served as a fixation point, presented at the beginning of each trial in the center of the screen, was set to 10% of the display height.

### Procedure

Task instructions were presented on-screen in white against a black background. Participants were asked to react to different words appearing in the center of the screen. There were four response keys—D, F, J, K—that were assigned to the left middle finger, left index finger, right index finger, and right middle finger, respectively. Each response option was always associated with two stimuli (e.g., D for “I” and “myself”; F for “me” and “my”; J for “he” and “she”; and K for “you” and “your”). We counterbalanced response assignments across participants such that half the participants responded to self-relevant targets using their left hand (and non-self-relevant targets with their right hand), and the remaining half responded to self-relevant targets with their right hand (and non-self-relevant targets with their left hand).

**Fig. 1:**
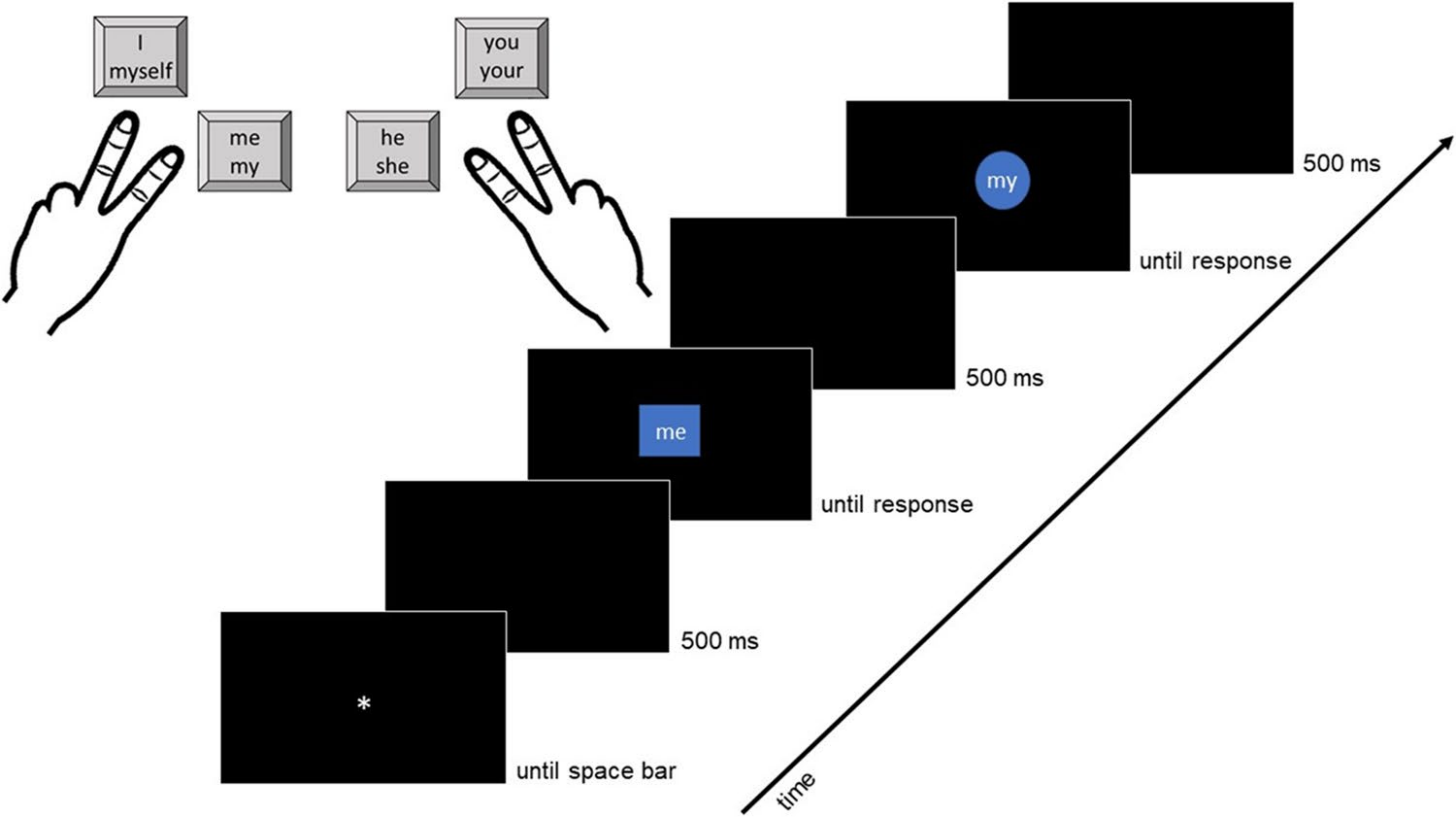
Example trial procedure and key assignment. *Note.* In this example trial, the geometric distractor shape changes between prime and probe. Although the target words change, participants should give the same response to prime and probe; thus, this is a response-repetition distractor-change (RRDC) trial

The trial sequence is illustrated in Fig. 1. Each trial began with a fixation point (an asterisk) presented centrally. Participants pressed the space bar to begin. After a 500-ms blank screen, the prime display was shown, comprising a word (i.e., the target) and a shape (i.e., the distractor). The display was shown in the center of the screen until a target response was given. After the response, the screen remained blank for 500 ms, and then the probe display appeared, comprising another target word and distractor shape; this was again presented centrally and remained on-screen until the participant responded. At the end of each trial, a further blank screen was shown for 500 ms.

The required target response was either repeated (response repetition; RR) or changed (response change; RC) between prime and probe. Also, the distractor was repeated (distractor repetition; DR) or changed (distractor change; DC) between prime and probe. This resulted in four different hypothesis-relevant trial types: response repetition and distractor repetition (RRDR), response repetition and distractor change (RRDC), response change and distractor repetition (RCDR), and response change and distractor change (RCDC). Each prime target appeared equally often with each geometric shape. In distractor-change trials, the probe distractor shape was randomly chosen from the three shapes that differed from the prime display.

Prime and probe targets were varied orthogonally, that is, each combination of prime and probe target appeared equally often. However, we defined three types of trials for the analysis: (1) we defined a trial as self-relevant if both prime and probe target were self-relevant (i.e., I, myself, me, my); correspondingly, (2) we defined a trial as other-relevant if both prime and probe target were other-relevant (i.e., he, she, you, your; see *Design*). These trials constituted our experimental trials. Of note, for our main analysis we disregarded target-repetition trials to obtain DRB effects that were not biased by target-repetition effects; however, in a supplementary analysis we calculated DRB effects with target-repetition trials included. Finally, (3) trials with a relevance change between prime and probe were treated as filler trials (serving mainly to disguise the design for participants), although they were used as a third set of trials in some exploratory analyses.

In summary, there were 512 trials (8 prime terms × 8 probe terms × 4 prime shapes × 2 [i.e., repetition vs. random change] probe shapes). Half of these trials (i.e., 256) were experimental trials, with each condition (i.e., RRDR, RRDC, RCDR, RCDC) implemented 64 times. The specific target was repeated in half the response-repetition trials; in the other half, prime and probe used different targets of the same relevance (i.e., self- vs. other-relevant). The remaining 256 trials were filler trials (i.e., there was a relevance change between prime and probe). These trials were always response-change trials, half of them with distractor repetition and half of them with distractor change.

Before the actual experimental phase, there was a practice phase (with 12 trials), during which participants received accuracy feedback after each response. During the experimental phase, participants received no feedback.

## Results

We used an alpha level of .05 (two-tailed) for all statistical tests, except for follow-up tests of DRB effects, which were one-tailed.

### Response times

Analysis focused on probe RTs from trials with correct answers to both prime and probe. Only RTs greater than 150 ms and below 1.5 interquartile ranges above the third quartile of the overall RT distribution (Tukey, 1977) were used for the analysis. Averaged across participants, 78% of trials were selected for RT analysis; 9% of trials were excluded because of erroneous prime responses, 7% because of erroneous probe responses, and 6% due to the RT-outlier criterion. Mean RTs and error rates are shown in Table 1. As already noted in the *Procedure* section, trials with target repetition were omitted from analyses (see Appendix for an analysis including these trials).

**Table 1:** Mean Probe Reaction Times (in ms; Error Rates in % in Parentheses) Across Conditions

		Distractor	
Relevance	Response	Change	Repetition
Self	Repetition	713 (0.34)	726 (0.31)
	Change	738 (0.50)	733 (0.51)
Other	Repetition	699 (0.19)	679 (0.20)
	Change	731 (0.27)	733 (0.30)
Relevance change from prime to probe			
Self to Other	Change	745 (0.74)	755 (0.84)
Other to Self	Change	819 (1.43)	823 (1.43)

The top panel shows RTs for trials in which relevance stays constant (with target-repetition trials excluded); the bottom panel shows RTs for relevance-change trials. In the top panel the standard errors of the mean (*SE*) for reaction times range from 12 to 16 ms for self-relevant trials (0.04–0.07 % for errors). *SEs* for other-relevant trials range from 11 to 13 ms (0.03–0.04 % for errors). In the bottom panel *SEs* for self to other trials range from 13 to 14 ms (0.09 % for errors), for other to self trials from 15 to 16 ms (0.16–0.17 % for errors)

**Fig. 2:**
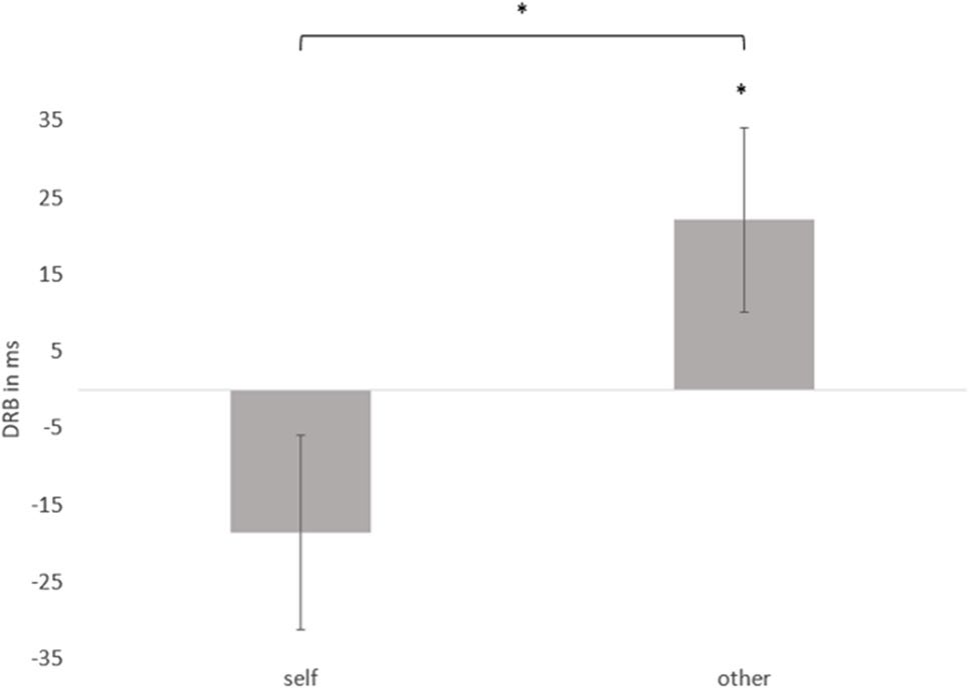
Mean Distractor–Response-Binding (DRB) Effects (in ms) as a Function of Relevance. *Note.* Error bars depict standard errors of the mean

We conducted a repeated-measures ANOVA with the factors distractor relation (*repetition* vs. *change*), response relation (*repetition* vs. *change*) and relevance (*self* vs. *other*). This analysis revealed significant main effects of response relation, *F*(1, 53) = 15.85, *p* < .001, η_p_^2^ = .23, participants responded faster in response repetition trials. There was also a main effect of relevance, *F*(1, 53) = 8.00, *p* = .007, η_p_^2^ = .13, participants responded faster in other-relevant trials compared to self-relevant trials. Moreover, the two-way interaction between relevance and response relation was significant, *F*(1, 53) = 5.89, *p* = .019, η_p_^2^ = .10. Neither the two-way interaction between relevance and distractor relation, *F*(1, 53) = 1.65, *p* = .205, η_p_^2^ = .03, nor the interaction between response relation and distractor relation, *F* < 1, reached significance. The crucial three-way interaction of distractor, response relation, and relevance was significant, *F*(1, 53) = 7.19, *p* = .010, η_p_^2^ = .12 (*d*_***z***_ = 0.36).^1^ Thus, the DRB effect was moderated by relevance (see Fig. 2).

We computed DRB effects as the distractor-repetition benefit in response-repetition trials minus distractor-repetition interference in response-change trials ([RRDC – RRDR] – [RCDC – [RCDR]), separately for both relevance conditions (see Fig. 2). We found significant distractor–response binding in trials without self-relevance, *M* = 22 ms (*SE* = 12 ms), *t*(53) = 1.86, *p* = .035, *d*_***z***_ = 0.25, but a numerically negative DRB index in self-relevant trials, *M* = -19 ms (*SE* = 13 ms).^2^

### Error rates

We conducted the same analyses for error rates. The ANOVA showed significant main effects of relevance, *F*(1, 53) = 21.82, *p* < .001, η_p_^2^ = .29, and response relation, *F*(1, 53) = 16.30, *p* < .001, η_p_^2^ = .24. The two-way interaction between relevance and response relation did not reach significance, *F*(1, 53) = 2.82, *p* = .099, η_p_^2^ = .05. Also, the other two-way interactions as well as the crucial three-way interaction were not significant, all *F*s < 1. We also calculated DRB effects for errors. Neither the DRB effect for self-relevant targets, *t*(53) = .62, *p* = .319, *d*_*z*_ = 0.09, nor for non-self-relevant targets, *t*(53) = .47, *p* = .269, *d*_*z*_ = 0.07, was significant.

### Relevance-change trials

Table 1 additionally shows the results for relevance-change trials. These trials may provide an indication of whether self-related moderation of DRB effects is dominantly located in the prime trial (i.e., change in binding processes) or the probe trial (i.e., change in retrieval processes). As can be seen, in one condition—that is, self-to-other change—there was a notable difference in RTs between distractor change and distractor repetition that fits the general DRB pattern; it was statistically significant, *t*(53) = 2.33, *p* = .012, *d*_*Z*_ = 0.32 (*t*[53] = 1.34, *p* = .093, *d*_*Z*_ = 0.18 for errors). The corresponding difference in other-to-self change trials is negligible, both |*t*|s < 1 for RTs and errors. The contrast between the change types was not significant, both |*t*|s < 1.04 for RTs and errors.^3^

However, the power of this contrast was quite low because the relevance-change trials provide only a “half DRB” effect because response-repetition trials are (structurally) missing. Therefore, we explored another approach to integrating these trials into the analyses: If we tentatively assume that the processes responsible for the relevance differences in the DRB effects are located in the probe trial (i.e., if we assume differences in retrieval processes), the DRB differences between self and other should be more pronounced if the other-to-self change trials are subsumed under the self-relevant category and the self-to-other change trials are added to the other-relevant category rather than the other way around. Indeed, defining relevance as *probe* relevance, we found significant distractor–response binding in trials without self-relevance, *M* = 26 ms (*SE* = 11 ms), *t*(53) = 2.45, *p* = .009, *d*_*z*_ = 0.33, but no significant distractor–response binding in self-relevant trials, *M* = −13 ms (*SE* = 12 ms), *t*(53) = −1.11, *p* = .136, *d*_*z*_ = 0.15. Most importantly, the DRB effects differed significantly from each other (the difference between both effects was *M* = 39 ms, *SE* = 13 ms), *t*(53) = 2.98, *p* = .002, *d*_*z*_ = 0.41. Compared with our main analysis (*d*_*z*_ = 0.36; see above), the contrast was thus slightly more pronounced.

If we define relevance as *prime* relevance, the two effects differ significantly from each other as well, *M* = 34 ms (*SE* = 15 ms), *t*(53) = 2.35, *p* = .011, *d*_*z*_ = 0.32, but relative to our main analysis (*d*_*z*_ = 0.36; see above), the effect was slightly smaller. DRB for trials without self-relevance was *M* = 23 ms (*SE* = 11 ms), *t*(53) = 2.13, *p* = .019, *d*_*z*_ = 0.29; DRB for self-relevant trials was *M* = -11 ms (*SE* = 12 ms), *t*(53) = -.86, *p* = .197, *d*_*z*_ = 0.12.

## Discussion

In the present study, we investigated how self-relevance modulates distractor–response-binding (DRB) effects, that is, the influence of distractor-based binding and retrieval on responding. Previous studies (e.g., Dignath et al., 2022; Golubickis & Macrae, 2021) assumed that self-relevance enhances executive control processes. Enhanced control processes should lead to a reduced influence of distractors on performance and should therefore reduce DRB effects in a top-down manner. In our experiment, we presented self-relevant versus non-self-relevant pronouns as targets and geometric shapes as distractors. Our data provided support for the hypothesis: If targets were self-relevant, no DRB effects were found, whereas in non-self-relevant trials, DRB effects emerged. Moreover, the DRB effects differed significantly from each other. Thus, our experiment provides some initial evidence on how self-relevance can influence the short-term binding of distractors to responses. The results support the notion that if a target stimulus has self-relevance, executive control processes are triggered, which not only reduce but can completely prevent processing of distractors.

The present findings provide further insight into how self-relevance influences our actions. Generally, it is assumed that self-relevant stimuli strengthen bindings (Sui & Humphreys, 2015a; Humphreys & Sui, 2016). For example, Sui and Humphreys (2015b) found a stronger redundancy gain (i.e., faster reaction times when two conceptually equivalent stimuli instead of one are presented) for self-relevant than for non-self-relevant stimuli. They hypothesized that two self-relevant stimuli are more strongly bound together in a common representation than non-self-relevant ones. This stronger binding of self-relevant stimuli then facilitates perceptual and action processes. Moreover, it has been shown that shapes that someone previously associated with the self are more difficult to associate with another person later on (Wang et al., 2016). Our results show that the self-relevant stimuli do not appear to nonspecifically enhance binding processes. When two stimuli (in our case a distractor and a target stimulus) occur in a joint event, the distractor is not bound more strongly to a response when the target stimulus is self-relevant. Otherwise, DRB effects should have been stronger for self-relevant trials. Thus, binding advantages for self-relevant stimuli do not appear generally.

As mentioned above, the BRAC framework (Frings et al., 2020) assumes that integration and retrieval are two independent processes that can be separately influenced by top-down or bottom-up mechanisms. It has recently been suggested that binding processes are relatively automatic processes that are influenced to a lesser extent by top-down or bottom-up mechanisms than retrieval processes (Frings & Rothermund, 2011; Hommel, 2005; Moeller & Frings, 2014). Retrieval is less automatic and thus more susceptible to be influenced by these mechanisms (Hommel et al., 2014; Ihrke et al., 2011; Laub et al., 2018; Moeller & Frings, 2014). Since our interpretation of the present results is that self-relevance increases executive control, it is plausible to assume that the relevance of target stimuli influences binding and retrieval processes to different extents. The present experiment cannot fully address this question because our main analyses only included trials in which both prime and probe target were either self-relevant or non-self-relevant. However, taking the trials with relevance changes between prime and probe into account, we can provide an estimate of whether target self-relevance has a stronger effect on distractor binding or distractor retrieval. Including these trials by defining relevance type as probe relevance, we found a more pronounced difference between self- and other-related DRB effects than in the standard analysis. By contrast, the difference between self- and other-relevant DRB effects were less pronounced if relevance was defined as prime relevance. This pattern suggests that self-relevance has a stronger influence on probe-retrieval than on prime-integration processes. This interpretation meshes well with previous research arguing that integration is more automatic and therefore less easily influenced (here by self-relevance) than probe retrieval (e.g., Hommel, 2005).

In our study, we used pronouns, that is, verbal materials, to manipulate the self-relevance of targets. Sui et al. (2013) demonstrated that the posterior superior temporal sulcus (pSTS) shows increased activation when self-relevant label-shape combinations are processed and that the pSTS is specifically also associated with the processing of self-relevant labels (see also Humphreys & Sui, 2016). Moreover, the pSTS has generally been associated with the processing of linguistic material (e.g., Wilson et al., 2018). Thus, our results may depend in particular on the involvement of the pSTS since subjects in our experiment process linguistic stimuli that may or may not be self-relevant. Other studies which investigate influences of self-relevance typically also use non-linguistic stimuli, which are associated with a person’s self or another entity (typically geometric shapes are used, but other stimulus types work as well; Schäfer et al., 2016). Further studies should investigate how the results are affected when non-linguistic stimuli (such as the geometric shapes just mentioned) are used and the pSTS is less involved in stimulus processing. A reduced activation of the pSTS could lead to a reduced influence of self-relevance, so that in turn the DRB effect might be less diminished. Yet, on the other hand, event-file dynamics underlying binding effects reflect processing in distributed cortical and subcortical networks encompassing a variety of brain areas (Beste et al., 2023). It was therefore argued that with respect to brain oscillations theta band activity (TBA; suitable to integrate information across spatial distances) reflects event-file configuration. Intriguingly, TBA has just recently be shown to also reflect processing of self-relevant stimuli in the SPE task (Haciahmet et al., 2023). The role of the pSTS for the influence on self-relevance on DRB effects remains thus an open question for future research.

We also would like to note the main effect of the relevance condition (both in RTs and errors) was due to slower reaction times respectively more errors in the self-relevant trials. When processing self-relevant stimuli, one usually expects faster reaction times and fewer errors for these trials (see, e.g., Sui et al., 2012). In this respect, it is noteworthy that participants in our study generally reacted more slowly when responding to self-relevant targets compared to other-relevant targets. However, this pattern does not contradict our finding that DRB effects are moderated by self-relevance. At this point we refrain from a more detailed interpretation of this result, since we do not really have a theoretical basis to explain this effect.

In summary, the present study shows that self-relevance has even more influence on stimulus processing as previously assumed—distractor-based binding is diminished if targets are self-relevant. We argue that this demonstrates that self-relevance increases executive control, which in turn modulates the dynamic control of distractor processing.

## Data Availability

During the review process, data from the experiment will be made available as Electronic Supplementary Material. In case of publication, the raw data and code books will be made publicly available at OSF (https://osf.io/3gkrp).

## Appendix

Because previous findings suggest that target repetition cannot fully explain DRB effects (Frings, Rothermund, et al. 2007; Frings, Wentura, et al. 2007, Exp. 2), we here additionally report DRB effects where we included target-repetition trials (see Table 2): The DRB effect for self-relevant targets was again numerically negative, *M* = −5 ms (*SE* = 9 ms). The DRB effect for non-self-relevant targets, *M* = 21 ms (*SE* = 9 ms), reached significance, *t*(53) = 2.39, *p* = .010, *d*_***z***_ = 0.33. Moreover, both effects differed significantly from each other, *t*(53) = −2.01, *p* = .049, *d*_***z***_ = 0.27. For error rates, there were no significant DRB effects for self-relevant targets, *t*(53) = .49, *p* = .314, *d*_***z***_ = 0.07, or other-related targets, *t*(53) = .27, *p* = .394, *d*_***z***_ = 0.04, *t*(53) = .23, *p* = .817, *d*_***z***_ = 0.03 for the difference.

**Table 2:** Mean Probe Reaction Times (in ms; Standard Deviations in Parentheses) Across Conditions

Relevance	Response	Distractor		
self	repetition	Change		639 (1.98)
		Repetition		638 (1.83)
	change	Change		738 (2.56)
		Repetition		733 (2.59)
other	repetition	Change		623 (1.15)
		Repetition		605 (1.26)
	change	Change		731 (1.37)
		Repetition		733 (1.56)

The table shows RTs for trials in which relevance stays constant (with target-repetition trials included). Including target-repetition trials did not change mean RTs for response-change trials because target repetition is always response repetition

## Footnotes

1 This *F* test equals a one-sample *t* test for the difference between the self-relevant DRB effect and the other-relevant DRB effect. The requirement for calculating a one-sample *t* test is that the corresponding variable is normally distributed. This assumption is violated here. Since this assumption is violated, we conducted a Wilcoxon rank test as an alternative. This test is also significant, *Z* = 2.36, *p* = .018, and thus leads to the same conclusion as the significant three-way interaction.

2 For the sake of transparency: the negative DRB effect was not significant in the appropriate two-tailed test, *t*(53) = −1.48, *p* = .146, *d*_*z*_ = 0.20. The test is not significant even if a one-sided test was adopted for reasons of equivalence with the test for the condition without self-relevance, *p* = .073.

3 Quite obvious from Table 1 is additionally a large main effect of relevance-change type (with self-to-other changes being faster and more accurate than other-to-self changes), *t*(53) = 9.27, *p* < .001, *d*_*Z*_ = 1.26 for RTs and *t*(53) = 4.27, *p* < .001, *d*_*Z*_ = 0.58 for errors. This effect was not expected and we refrain from speculating about its causes.
